# Serum and Seminal Plasma Levels of Lead and Arsenic in Cigarette Smokers and Their Relation to the Semen Parameters

**DOI:** 10.1007/s12011-023-04039-7

**Published:** 2024-01-05

**Authors:** Azza Gaber Antar Farag, Eman Abd-Elfatah Badr, Alaa Osama Ali Kholif, Mostafa Nabil Khalifa, Mai Medhat Mohamed Ghanem

**Affiliations:** 1 Dermatology, Andrology and STDs department, Faculty of Medicine, Menoufia University, Shebin El-Kom, Egypt; 2 Medical Biochemistry and Molecular Biology Department Faculty of Medicine, Menoufia University, Shebin El-Kom, Egypt; 3 Birket El Sabe, 32661 Menoufia Egypt

**Keywords:** Arsenic, Lead, Semen parameters, Smoking

## Abstract

Male infertility along with altered semen parameters have been related to smoking. Smoking-related elevations in serum and seminal lead (Pb) and arsenic (As) may play a role in mediating the toxic effects of smoking on seminogram. This research aims to determine whether smoking has any significant impact on Pb and As levels in the seminal plasma and serum, as well as on the various semen parameters, when compared to nonsmokers. In total, 80 adult males were included: 60 smokers and 20 age-matched nonsmokers. Based on the number of cigarettes smoked/day (CPD), the smokers were categorized into mild (1–10), moderate (11–20), and severe (> 20). The analysis of semen was conducted in accordance with the 2010 WHO laboratory manual. Using an atomic absorption spectrophotometer, Pb and As concentrations in the serum and seminal plasma of all groups were determined. Compared to nonsmokers, smokers had a significantly reduced sperm count, motility, and viability, as well as a larger percentage of aberrant forms (*P* = 0.001, 0.025, 0.034, 0.002 respectively). Smokers had higher Pb concentrations in their serum and seminal fluid than nonsmokers (*P* = 0.002, 0.001 respectively). Seminal Pb had a significant negative correlation with sperm count (*P* = 0.004, r = -0.320). Serum Pb levels were found to positively correlate with seminal Pb levels (*P* 0.001, r = 0.648), and cigarette smokers had substantially greater seminal As levels than nonsmokers (*P* = 0.024). Sperm viability was strongly inversely related to seminal As (*P* = 0.042, r = -0.264). Seminal As levels and aberrant sperm shapes were found to be significantly correlated (*P* = 0.001, r = 0.414). In smokers, a significant positive relationship between seminal As and seminal Pb was observed. Therefore, semen parameters could be adversely affected by smoking through high levels of Pb and As (*P* = 0.012, r = 0.298).

## Introduction

Smoking is one of the most common behaviors among men, especially those of reproductive age [1]. Cigarette smoking can have toxic effects on spermatogenesis either directly or indirectly [2]. Tobacco smoke contains over 4,000 compounds, some of which are heavy metals like Pb and As [3].

Pb, a versatile and ubiquitous metal, has been used since prehistoric times. It is now ubiquitous and has been mobilized in the environment [4]. Multisystemic, deleterious effects of chronic Pb exposure occur via a variety of biochemical processes. Heavy Pb exposure has been related to various health problems, such as anemia, hypertension, brain and kidney damage, and premature death in both adults and children. Also, researchers discovered that male fertility declines with prolonged exposure to Pb [5].

Pb accumulation was observed in the testis and epididymis as the concentration increased, accompanied by a decrease in the activities of alkaline phosphate and Na + /K + -ATPase, both of which are known to be involved in spermatogenesis and spermiogenesis, respectively. Seminiferous tubule lumen accumulation of immature cells, complete cessation of spermatogenesis, and epididymal basement membrane injury were observed as histological alterations induced by higher concentrations of Pb [6]. An overproduction of inhibin B has been related to Sertoli cell dysfunction caused by high levels of Pb exposure, which may explain the decreased levels of follicular stimulating hormone (FSH) [7].

Toxic forms of the element As can be found throughout nature. Gastroenteritis, neurological symptoms, vascular alterations, diabetes, and various malignancies (including those of the bladder, lung, liver, kidney, and prostate) are just a few of the health problems related to As exposure [8, 9]. The male reproductive system may be negatively impacted by As due to its direct effects on the testis. Eventually, this produces a disruption in the neuroendocrine system and hormone production, which has repercussions for spermatogenesis [10].

The objective of this study was to determine the potential correlation between the consumption of cigarettes and the amounts of Pb and As found in serum and seminal plasma.

## Materials and Methods

This case–control study was performed on 80 married individuals, ages ranging from 30 to 50 years old, attending the andrology clinic at our institution. These individuals were classified into two groups. Group 1 included 60 smokers who had smoked for more than one year and was subdivided into three subgroups, each with 20 smokers according to the number of cigarettes per day (CPD): mild (1–10 CPD), moderate (11–20 CPD), severe (> 21 CPD) [11]. Group 2 included 20 healthy nonsmoker volunteers as a control group.

We excluded patients with occupational exposure to Pb and/or As. Moreover, patients with drug intake or who had operations that may affect semen parameters were excluded. Subjects with abnormal genital examination, abnormal sex hormones, and abnormal secondary sexual characters were also excluded. Each participant underwent a comprehensive review of their personal, marital, and medical history. There was both a general and local examination.

### Laboratory Investigation

Approximately 5 mL of blood was withdrawn from each participant under aseptic conditions, left to clot for half an hour, then centrifuged at 3000 rpm/min for 15 min, and the serum was kept at -20 °C until the serum was digested for determination of Pb and As concentrations. Serum digestion procedure was performed by adding 2 mL of nitric acid (70%) and 1 mL of perchloric acid (70%) to 0.5 mL of serum in a Pyrex tube and then heating this compound using a water bath over a hot plate. Then, the mixture was placed at 160 °C for 1 h and cooled; it was finally completed to 10 mL with (30%) hydrochloric acid. This compound was also heated with a water bath on a hot plate at 160 °C ​in order to obtain the absolute digestion of serum and vaporization of acids [12].

Collection of semen was done by masturbation into a sterile cup after 2–5 days of abstinence. Semen analysis was conducted using computer-assisted semen analysis. Ejaculate volume and liquefaction time were measured. In accordance with WHO standards 2010, sperm count, viability, motility, and morphology were evaluated after sperm samples were melted at 37 °C [13]. Then, isolation of seminal plasma was done and kept at -20 °C. Moreover, 100 μl of seminal plasma was digested with 500 μl of super-grade 0.8 M HNO_3_ in a glass tube. The residue of digested seminal plasma and serum was dissolved in 1 ml of 1% HNO_3_ and applied to a graphite tube for detection of Pb and As using an atomic absorption spectrophotometer (Varian SpectrAA 200Z, USA). The recovery of Pb in spiked semen and serum samples was 97%, respectively. The instrument was calibrated using 5, 10, and 15 μg/l standards for Pb. A sample blank was prepared with each set of samples to control for possible metal contamination from external sources. The level of detection for Pb was 0.1 μg/l. Calibration curves for As concentrations of 0, 0.1, 0.5, 1, 5, 10, 20, and 50 μg L-1 were analyzed. The standard reference (0.1 and 1 ppb) was injected into every tenth sample to assess instrumental sensitivity and stability. Furthermore, the calibration blank was measured to assess any background carryover, and duplicate samples were analyzed to observe reproducibility during analysis. The limit of detection was 0.2 μg/l.

### Statistical Analysis

Data were analyzed on an IBM PC with SPSS version 19 (SPSS, Inc., Chicago, Illinois, USA). Mean (X-), standard deviation (SD), range, and numbers and percentages were used to represent quantitative and qualitative data. A chi-square test (χ2) was employed to examine the correlation between two qualitative variables. Mann–Whitney test was used to compare two nonnormally distributed quantitative groups. The relationship between quantitative and qualitative ordinal variables was measured using Spearman’s correlation (r). A *P* value ≤ 0.05 signifies statistical significance.

## Results

Age, duration of marriage, number of children, and age of youngest kid were not significantly distinct among smokers and nonsmokers. Intercourse frequency was significantly lower in smokers than in nonsmokers, indicating a significant variation between the two groups (Table 1).

**Table 1:** Comparison between smokers and nonsmokers regarding their demographic data

Studied variables	Smokers (*N* = 60)	Non smokers (*N* = 20)	t-test	*P* value
Age / years				
Mean ± SD	37.6 ± 4.30	37.8 ± 5.55		
Median	37.0	36.5	0.139	0.890
Range	30.0 – 49.0	30.0 – 47.0		
Period of marriage (years)				0.198
Mean ± SD	9.98 ± 5.63	8.05 ± 5.02	U	
Median	8.50	6.50	1.28	
Range	1.00 – 24.0	2.00 – 17.0		
Number of children				
None	4(6.70)	2(10.0)		
One	13(21.7)	8(40.0)	χ2	
Two	22(36.7)	3(15.0)	5.51	0.238
Three or more	18(30.0)	7(35.0)		
Age of youngest child (years)				
Mean ± SD	5.35 ± 3.11	4.27 ± 2.10	U	
Median	5.00	4.00	1.10	0.269
Range	1.00 -10.0	1.00 – 8.00		
Frequency of intercourse / week				
One	23(38.3)	0(0.00)	χ2	< 0.001*
Two	27(45.0)	6(30.0)	22.7	
Three	10(16.7)	14(70.0)		

SD: Standard deviation, significance level at P value < 0.05 χ2: Chi squared test U:Mann Whitney test

There were no significant differences between smokers and nonsmokers in terms of semen volume, liquefaction time, or pus cell count (*P* > 0.05 for all). Sperm count and motility were significantly lower in smokers than those in nonsmokers (34.3 ± 28.4 million/ml vs. 70.4 ± 27.9 million/ml and 31.7 ± 16%, vs. 41.1 ± 14.3%, *P* = 0.001 and 0.025, respectively). Sperm motility was significantly higher in mild smokers than in moderate and severe smokers (39.5 ± 13.5%, vs. 27.4 ± 17.1%, and 28.2 ± 15.4%; *P* = 0.017 and 0.016, respectively). Smokers had significantly lower sperm viability than nonsmokers (55.1 ± 19.7 vs. 65.5 ± 15.2%; *P* = 0.034). Severe smokers showed significantly lower sperm viability compared to mild smokers (45.7 ± 19.3%, vs. 64.5 ± 16.9%; *P* = 0.011). In smokers, the percentage of abnormally formed sperm was significantly higher than in nonsmokers (50.0 ± 19.3%, vs. 34.8 ± 15.2%; *P* = 0.002). In severe smokers, the percentage of abnormally formed sperm was significantly higher than in mild and moderate smokers (61.3 ± 14.9%, vs. 39.7 ± 17.9%, and 48.9 ± 19.2%, *P* = 0.001 and 0.022, respectively) (Table 2).

**Table 2:** Comparison between smokers and nonsmokers regarding seminal parameters

Studied variables	Smokers (*N* = 60)	Smoker groups			Non smokers (*N* = 20)	Test of sig *P* value	Post hoc test	Test of sig	*P* value
		Mild (*N* = 20)	Mod. (*N* = 20)	Severe (*N* = 20)					
Volume (ml) Mean ± SD Range	2.46 ± 1.32 0.50 – 7.00	2.62 ± 1.22 1.00 – 5.00	2.54 ± 1.78 0.50 – 7.00	2.22 ± 0.81 1.00 – 4.00	2.42 ± 0.89 1.00 – 4.00	K 1.21 0.749	P1:0.443 P2: 0.357 P3: 0.742 P4:0.923 P5:0.537 P6:0.474	U .411	0.681
L.T.(min) Mean ± SD Range	25.5 ± 6.04 15.0 – 40.0	25.7 ± 7.65 15.0 – 40.0	26.2 ± 3.93 20.0 – 30.0	27.5 ± 6.97 15.0 – 40.0	27.0 ± 3.76 20.0 –30.0	K 1.79 0.617	P1:0.403 P2:0.553 P3:0.617 P4:0.673 P5:0.525 P6:1.00	U 1.33	0.182
Count (million/ml) Mean ± SD Range	34.3 ± 28.4 2.00 – 135.0	42.0 ± 35.5 7.00 – 135.0	25.6 ± 18.3 2.50 – 70.0	35.4 ± 27.8 2.00 – 100.5	70.4 ± 27.9 35.0 – 160.0	K 23.5 0.001*	P1:0.148 P2:0.715 P3:0.002* P4:0.358 P5:0.001* P6:0.001*	U 4.61	0.001*
Motility (%) Mean ± SD Range	31.7 ± 16.1 0.45 – 65.0	39.5 ± 13.5 10.0 – 60.0	27.4 ± 17.1 2.13 – 60.0	28.2 ± 15.4 0.34 – 65.0	41.1 ± 14.3 10.0 – 73.0	K 12.5 0.006*	P1:0.017* P2:0.016* P3:0.903 P4:0.787 P5:0.012* P6:0.008*	t-test 2.29	0.025*
Viability (%) Mean ± SD Range	55.1 ± 19.7 13.2—96.0	64.5 ± 16.9 35.0—96.0	55.0 ± 19.0 13.2 – 82.0	45.7 ± 19.3 14.3 – 75.0	65.5 ± 15.2 43.0 – 90.0	K 11.1 0.011*	P1:0.184 P2:0.011* P3:0.724 P4:0.123 P5:0.088 P6:0.004*	t-test 2.16	0.034*
Abnormal forms (%) Mean ± SD Range	50.0 ± 19.3 13.0 – 95.2	39.7 ± 17.9 13.0 – 70.0	48.9 ± 19.2 20.0 – 95.2	61.3 ± 14.9 40.0 – 86.1	34.8 ± 15.2 16.0 – 76.-	K 22.1 0.001*	P1:0.133 P2:0.001* P3:0.432 P4:0.022* P5:0.010* P6:0.001*	U 3.11	0.002*
Pus cells (HPF) Mean ± SD Range	3.25 ± 1.85 1.00 -7.00	3.50 ± 1.63 1.00 – 7.00	3.05 ± 2.21 1.00 – 7.00	3.20 ± 1.73 1.00 – 7.00	3.25 ± 1.61 1.00 – 6.00	K 0.543 0.909	P1:0.260 P2:0.465 P3:0.582 P4:0.473 P5:0.433 P6:0.804	U 0.197	0.843

L.T: liquefaction time SD: Standard deviation U: Mann Whitney K: Kruskal Wallis test significance level at *P* value < 0.05 **P1:** Comparison between mild smokers and moderate smokers **P2**: Comparison between mild smokers and severe smokers **P3**: Comparison between mild smokers and non-smokers **P4**: Comparison between moderate smokers and severe smokers **P5**: Comparison between moderate smokers and non-smokers **P6**: Comparison between severe smokers and non-smokers

**Fig. 1:**
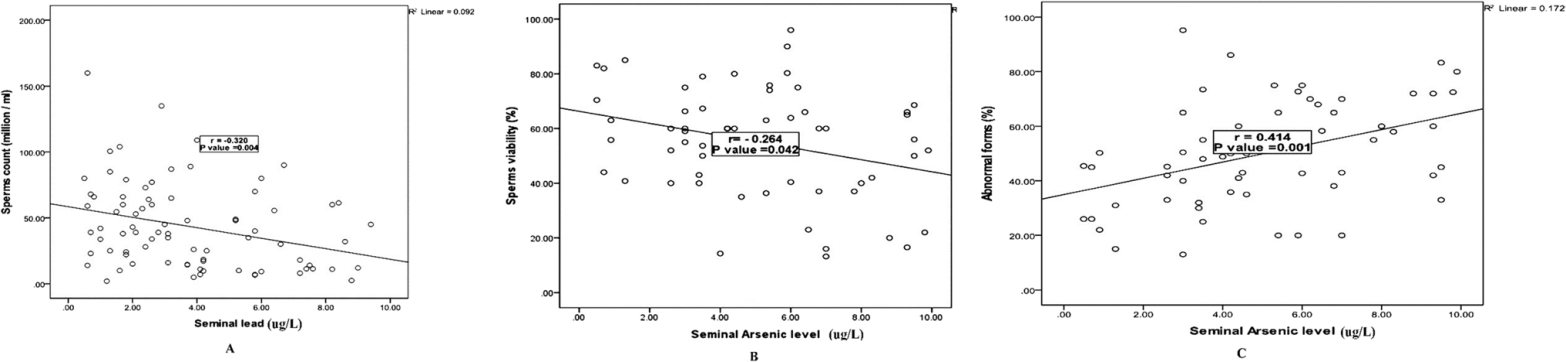
Correlation between seminal Pb & As levels and semen parameters. **a** Correlation between seminal Pb level and sperm count (million/ml) among smokers. **b** Correlation between seminal As level and sperm viability (%) among smokers. **c** Correlation between seminal As level and abnormal forms (%) of sperms among smokers

Smokers had substantially higher levels of seminal Pb than nonsmokers (4.85 ± 4.81 ug/L vs. 2.25 ± 1.47 ug/L; *P* = 0.001). Moreover, serum Pb level was significantly higher in smokers than nonsmokers (16.4 ± 12.8 µg/dL vs. 8.05 ± 3.41 µg/dL; *P* = 0.002). There were no significant differences in seminal and serum Pb regarding different grades of smoking (Table 3). Seminal Pb was significantly inversely related to sperm count (*P* = 0.004; r = -0.320) (Fig. 1a). However, there were nonsignificant correlations between seminal or serum Pb levels and age, number of CPD, and other semen parameters (*P* > 0.05 for all).

**Table 3:** Comparison between smokers and nonsmokers regarding their seminal and serum lead and arsenic levels

Studied variables	Smokers (*N* = 20)	Smoker groups			Non smokers (*N* = 20)	Test of sig	*P* value	Mann Whitney test	*P* value
		Mild (*N* = 20)	Moderate (*N* = 20)	Severe (*N* = 20)					
Seminal lead (ug/L) Mean ± SD Median Range	4.85 ± 4.81 4.00 0.60 – 37.0	3.58 ± 1.70 3.10 1.60 – 8.20	5.08 ± 2.73 5.50 0.70 – 9.00	5.48 ± 4.86 3.95 0.60 – 9.40	2.25 ± 1.47 2.05 0.50 – 5.60	K 13.1 0.004*	P1:0.056 P2:0.685 P3:0.007* P4:0.317 P5:0.001* P6:0.038*	3.31	0.001*
Serum lead (µg/dL) Mean ± SD Median Range	16.4 ± 12.8 10.6 1.60 – 63.0	11.4 ± 5.22 8.50 5.70 – 22.4	15.7 ± 10.7 14.2 1.60 – 40.0	21.9 ± 17.6 21.2 1.60 – 63.0	8.05 ± 3.41 6.70 3.50 – 15.0	K 10.3 0.016*	P1:0.552 P2:0.304 P3:0.007* P4:0.218 P5:0.027* P6:0.011*	3.05	0.002*
Seminal arsenic (ug/L) Mean ± SD Median Range	6.44 ± 2.36 6.45 2.30 – 9.90	4.28 ± 2.60 3.85 0.50 – 9.50	6.62 ± 2.27 6.45 2.60– 9.90	6.44 ± 2.36 6.45 2.30 – 9.90	4.27 ± 2.60 3.85 0.50 – 9.50	K 13.8 0.003*	P1:0.007* P2:0.012* P3:1.00 P4:0.828 P5:0.007* P6:0.012*	2.25	0.024*
Serum arsenic (ug/L) Mean ± SD Median Range	6.42 ± 1.82 6.70 2.40 – 9.40	6.79 ± 1.76 7.35 3.90 – 89.40	5.67 ± 1.79 5.95 2.40 – 9.00	6.79 ± 3.12 7.35 3.90 – 9.40	6.05 ± 1.97 5.95 2.40 – 9.60	K 6.63 0.085	P1:0.060 P2:1.00 P3:0.163 P4:0.060 P5:0.935 P6:0.163	0.957	0.339

SD: Standard deviation K: Kruskal Wallis test significance level at *P* value < 0.05

P1: Comparison between mild smokers and moderate smokers

P2: Comparison between mild smokers and severe smoker

P3: Comparison between mild smokers and non-smokers

P4: Comparison between moderate smokers and severe smokers

P5: Comparison between moderate smokers and non-smokers

P6: Comparison between severe smokers and non-smokers

Seminal As level was significantly higher in smokers than nonsmokers (6.44 ± 2.36 ug/L vs. 4.85 ± 2.36 ug/L; *P* = 0.024). Moreover, seminal As level was substantially lower in mild smokers than in moderate and severe smoker groups (4.28 ± 2.60 ug/L vs. 6.62 ± 2.27 ug/L and 6.44 ± 2.36 ug/L). There was no significant difference among the studied groups concerning serum As level (*P* = 0.339) (Table 3). Seminal As had a significant negative correlation with sperm viability (*P* = 0.042; r = -0.264) (Fig. 1b). And seminal As and sperm abnormal forms had a significant positive correlation (*P* = 0.001; r = 0.414) (Fig. 1c). However, there were nonsignificant correlations between seminal or serum As levels and age, number of CPD, and other semen parameters (*P* > 0.05 for all) (data not shown).

**Fig. 2:**
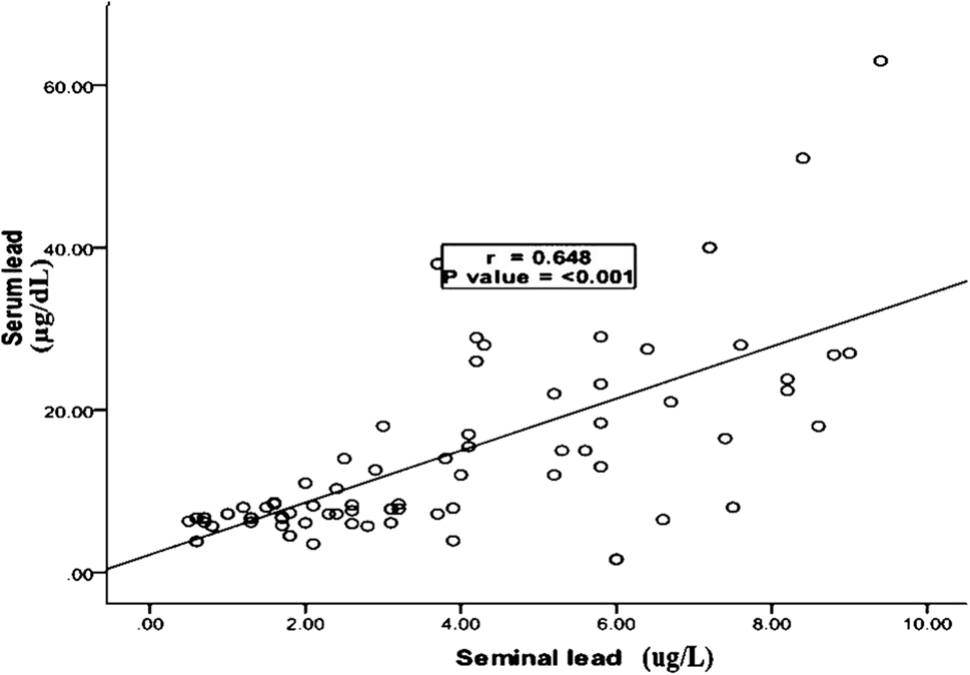
Correlation between seminal Pb level and serum Pb level among the smokers group

**Fig. 3:**
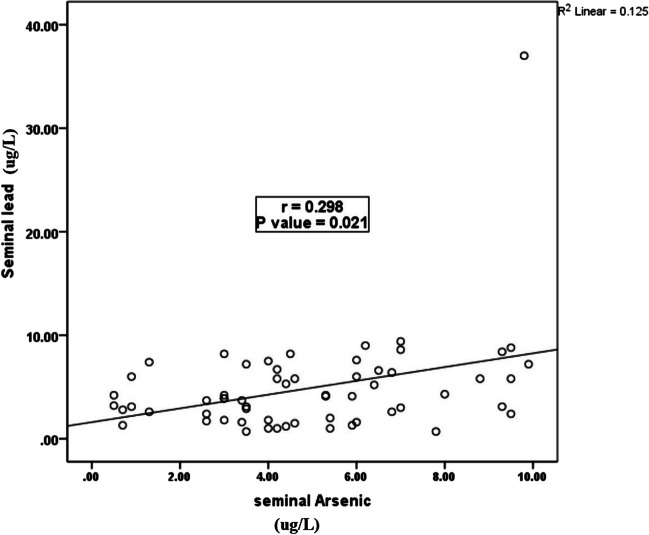
Correlation between seminal AS and seminal Pb levels among the smokers group

A substantial positive association was observed between the levels of Pb in the serum and Pb in the seminal fluid (*P* = 0.001; r = 0.648) (Fig. 2). However, a significant positive relationship between the levels of Pb and As in seminal fluid was observed (*P* = 0.012, r = 0.298) (Fig. 3).

## Discussion

Statistical analysis of the findings indicated that there was a significant decrease in the frequency of intercourse among smokers than nonsmokers. This observation is in agreement with the results of Weber et al. [14]. To explain this result, Biebel et al. revealed that cigarette smoking is one of the risk factors that might lead to erectile dysfunction [15].

As previously reported [16–18], we observed in this study that the smokers had significantly lower sperm density, percent of actively motile sperms and sperms viability as well as higher percent of abnormal forms compared to non-smokers. However, Pasqualotto et al. did not find any relationship between smoking and different semen parameters other than a decrease in semen volume. The authors suggested that the decline in semen volume may precede alterations in motility, sperm concentration, and morphology as an early indicator of poor semen quality [19].

Concerning seminal volume and liquefaction time, other studies were found to be in agreement with our results [20, 21]. However, Fawzy et al. reported a significant delay of liquefaction among smokers compared to nonsmokers, which could be due to their inclusion of both shisha and cigarette smokers [22].

How does cigarette smoking affect semen parameters? It could be due to the hormonal changes, the toxic content found in cigarette smoking that might have harmful effects on male germ cells, elevated reactive oxygen species, and decreased scavenging antioxidant activity in the seminal plasma of smokers [23]. Also, a decrease in essential elements and elevated levels of toxic heavy metals in all biological fluid may have significant negative effects on human reproductive health through their adverse impacts on the physiological and pathological functions [24, 25].

Compared to nonsmokers, smokers had significantly higher serum and seminal Pb levels, with a significant positive correlation between the two. This is consistent with the findings by other authors who concluded that Pb contaminates the smoke from cigarettes since it is burned up with the rest of the cigarette. Due to their small size, smoke particles can lodge in the most remote parts of the lungs and be absorbed directly into the bloodstream. It seems that no genital tract barrier can prevent Pb from entering the male reproductive tract [3, 26–30]. This was supported by the results reported by Debnath et al., who found a positive correlation between semen and serum Pb [31]. Further research discovered insignificant variations in serum and seminal Pb levels between smokers and nonsmokers, possibly because both were occupationally exposed to Pb [32–34].

Furthermore, in the smoker group, there was no correlation between both serum and seminal Pb levels and CPD. This was in agreement with Fatima et al., who stated that smokers did not show any significant difference in their semen Pb levels depending on the number of CPD [30]. However, Hsu et al. stated that both smokers and nonsmokers may have been exposed to Pb at work, which could account for the correlation between daily cigarette consumption and blood Pb levels.

Concerning the correlation between the seminal plasma Pb and the main items of semen parameters, the research demonstrated that seminal Pb is significantly negatively correlated to sperm count only and not to other semen parameters. However, previous studies are controversial and showed wide variations. To the best of our knowledge, one research reported a connection between Pb content in seminal plasma and only sperm count, which is statistically significant [35]. Rather than being the result of altered hypothalamic-pituitary function, the association between Pb concentration in sperm or sperm count could be due to a direct detrimental effect of Pb on spermatogenesis [36]. Many other studies reported that a statistically significant inverse association exists between the Pb concentration in seminal plasma and the count, motility, and morphology of sperm [37–39]. However, other studies failed to report any relationship between seminal plasma Pb and semen parameters, which may be attributed to the fact that these studies were conducted on a smaller number of individuals [31, 40–42].

Regarding As concentrations, the present study revealed a significantly higher seminal plasma (not serum) As level detected in smokers than non smokers. Also, Souza et al. and Nunzio et al**.** reported the same result [43, 44]. The authors concluded that the testes and the accessory organs may concentrate As in the seminal plasma. This may explain its elevation in the seminal plasma and not in serum in our investigated smoker males. However, Khoramdel et al. reported that there were correlations between high serum As level and abnormality of sperm morphology and a decrease in sperm chromatin condensation. Khoramdel et al.^’^s result can be explained due to exposure of their cases to different sources of As as drinking groundwater. Furthermore, genetic predisposition may play a role [45].

The seminal plasma As levels in smokers are significantly negatively correlated to sperm viability and positively correlated to the percentage of abnormal forms of sperm. Many authors were found to agree with these results and reported that infertile individuals may have decreased sperm viability due to As exposure, which might have affected sperm morphology and cytoskeletal structure [46–48].

In agreement with Nandi et al. [47], we noted that there was a significant positive relationship between seminal Pb and As levels. These both heavy metals (Pb and As) may have a singular or synergistic effect on seminal parameters. More research is required to explain the molecular mechanisms underlying the deleterious effects caused by these agents on reproductive function and spermatozoal integrity.

Also, Khoramdel et al. revealed that there was an association between these elevated serum Pb and As levels and poor sperm count, aberrant morphology, and impaired sperm motility. Pb and As concentrations in the serum of infertile men were significantly greater than those of fertile men. Pb and As concentrations in the serum of fertile men were substantially lower. This may be because histone-to-protamine replacement is significantly reduced in the sperm of infertile males, which reduces chromatin condensation and leads to infertility [45].

There is no well-defined mechanism for how heavy metals cause harm to male reproductive systems. Animal research suggests that these xenobiotics may limit androgen biosynthesis in Leydig cells, cause lipid peroxidation of the cell membrane, and contribute to oxidative destruction of DNA by inducing mitochondrial dysfunction, increasing free radical generation, or decreasing antioxidant levels. Increased morphological abnormalities, decreased motility, and reduced fertilization potential are likely all interrelated aspects of spermatozoa health [49].

## Conclusion

Semen parameters are affected adversely by smoking through high levels of heavy metals like Pb and As. These detrimental effects are related to the cumulative effect of smoking.

## Data Availability

No datasets were generated or analysed during the current study.
